# Chemical composition, antimicrobial and antioxidant activities data of three plants from Tunisia region: *Erodium glaucophyllum, Erodium hirtum and Erodium guttatum*

**DOI:** 10.1016/j.dib.2018.07.005

**Published:** 2018-07-10

**Authors:** Gadhoumi Hamza, Ben-Halima Emna, Walid Yeddes, Zohra Dhouafli, Tounsi S. Moufida, Hayouni El Akrem

**Affiliations:** a Faculty of Sciences of Tunis, Université of Tunis El Manar, Tunis, Tunisia; b Laboratory of Aromatic and Medicinal Plants, Center of Biotechnology at the Ecopark of Borj-cédria, BP-901, 2050 Hammam-Lif, Tunisia; c Laboratory of Extremophile plants, Center of Biotechnology at the Ecopark of Borj-cédria, BP-901, 2050 Hammam-Lif, Tunisia

**Keywords:** *Erodium glaucophyllum*, *Erodium hirtum*, *Erodium guttatum*, Biochemical composition, Antioxidant activity, Antimicrobial activity

## Abstract

In the present work, phytochemical contents (total phenolic content, total flavonoids, and condensed tannins), antioxidant potentials, and antimicrobial activities of three plants in the Mediterranean genus Erodium (*Erodium glaucophyllum*, *Erodium hirtum*, and *Erodium guttatum*) from the Tunisia region were analyzed. The results showed that *E. glaucophyllum* contained high levels of polyphenols, flavonoids, and tannins. Therefore, *E. glaucophyllum* possesses high antioxidant activities (2,2-diphenyl-1-picrylhydrazyl and ferric reducing antioxidant power scavenging activities), and high inhibition of linoleic acid oxidation. All three plants exhibited high antimicrobial activities. This study highlights tree plants’ importance as dietary sources for natural antioxidants can be used in traditional medicine and the pharmaceutical industry.

**Specifications Table**

**Table:** 

**Subject area**	Biology
**More specific subject area**	Medicinal plants chemical composition and activity
**Type of data**	Table, figure
**How data was acquired**	Three plants as dietary sources of natural antioxidants, and anti-microbial activity.
**Data format**	Analyzed
**Experimental factors**	Chemical composition, antimicrobial and antioxidant activities of *Erodium glaucophyllum, Erodium hirtum and Erodium guttatum*
**Experimental features**	Biochemical composition and anti-microbial activity.
**Data source location**	The samples were collected from Tunisia region
**Data accessibility**	With this article

**Value of the data**

• Data presented here provide Chemical composition, antimicrobial and antioxidant activities of *Erodium glaucophyllum, Erodium hirtum and Erodium guttatum.*
• Determination of the phytochemical content, total phenolic compounds, total flavonoids, and condensed tannins.
• Evaluation of the antioxidant power and antimicrobial activity of the three plants.
• Results have also important role sources of natural antioxidants, and might be appropriate for the development of reliable index to estimate tuber richness with bioactive molecules.

## 1 Data

Results indicated in Table 1 showed that the three plants contained high levels of polyphenols: 124±6 mg GAE/ml, 180±4.02 mg GAE/ml and 248.08±2 mg GAE/ml for *Erodium guttatum, Erodium hirtum* and *Erodium glaucophyllum*, respectively. In addition the plant showed high levels of flavonoids with *E. glaucophyllum* being the richest one (91.97±1.56 mg RE/ml). Concerning the levels of tannins they were 20±0.5 mg TA/ml, 42±1.3 mg TA/ml and 31.87±0.38 mg TA/ml, for *E. guttatum*, *E. hirtum* and *E. glaucophyllum*, respectively. In parallel, antioxidant activities of the plants were investigated namely radical DPPH scavenging activities, the reducing power and inhibition of the peroxidation of linoleic acid. Furthermore, the *E. glaucophyllum* process high antioxidant activity (Table 1).

**Table 1:** Reducing power, DPPH radical scavenging activity, and inhibition of the peroxidation of linoleic acid activity.

**Extracts**	Total phenolics (mg GAE/g DR*)	Flavonoids (mg RE/g DR**)	Condensed tannins (mg CE/g DR***)	**Reducing power (IC**_**50**_**µg/ml)**	**DPPH (IC**_**50**_**; µg/ml)**	**inhibition of the peroxidation of linoleic acid (IC**_**50**_**; µg/m)**
*Erodium hirtum*	180±4.02	63±4.1	42±1.3	16.3±2	49.1±3.6	42.5±4.2
*Erodium guttatum*	124±6	52±2.3	20±0.5	28.1±1.8	56.9±3.3	71.03±9.3
*Erodium glaucophyllum*	248.08±2	91.97±1.56	31.87±0.38	14.98±1.26	20.29±2.64	37.22±2.36
Vit C				13.15±1.65	5.18±1.98	–
AG					–	13.18±1.21

Results are expressed as mean of 3 experiments±SD.

* mg GAE/g DR: mg gallic acid equivalents per g dry residue.

** mg RE/g DR: mg of rutin equivalent per gram dry residue.

*** mg CE/g DR: mg catechin equivalent per gram dry residue.

In addition, the results (Table 2) showed that the three plants exhibit high antimicrobial activity. Furthermore, the *E. guttatum* possess high antimicrobial activity compared to *E. glaucophyllum*, and *E. hirtum*. These results show a high correlation between polyphenol content and antimicrobial activity. As a potential drug, three plants needs to be explored properly following bioactivity-directed fractionation in order to isolate bioactive constituents and to evaluate its therapeutic effects.

**Table 2:** Antimicrobial activity of tree plants extracts.

**Bacteria**	**Gram**	**Inhibition zone (mm)**			
		***Erodium glaucophyllum***	***Erodium guttatum***	***Erodium hirtum***	***Streptomycin***
*Escherichia coli 25922 ATCC*	Gram (−)	16.4±2	8.2±1.7	5.3±1	28.2±4
*Escherichia coli 8739 ATCC*	Gram (−)	14.4±3	5.7±1	2.7±0.5	30.2±2.5
*Staphylococcus aureus 25923 ATCC*	Gram (+)	9.1±1	6.4±2.6	8.2±2.5	25.4±2
*Serratia marcescens (Enterobacteriaceae) 13880 ATCC*	Gram (−)	5.2±0.9	3.9±1.2	2.1±0.5	24.1±3
*Enterococcus aerogenes ATCC (13048)*	Gram(−)	11.6±2	6.7±2.3	3.6±1	26.6±1
*Enterococcus faecalis 29212 ATCC*	Gram (+)	10.9±3	8.1±2	7.4±2	23.5±3
*Pseudomonas aeruginosa 27853 ATCC*	Gram (−)	2.4±1	–	–	18.1±2

Results are expressed as mean of 3 experiments±SD.

## 2 Experimental design, materials, and methods

### 2.1 Material

The tuber of the three plants of *E. glaucophyllum, E. hirtum* and *E. guttatum* were collected from Jebel Orbata National Park-Gafsa- Tunisia regions. Fifty grams of leaf powder is extracted by maceration in a volume of 400 ml of a water–methanol solution (50%, v/v), for 24 h and with continuous stirring. All strains were obtained from Laboratory of Extremophile plants, Center of Biotechnology at the Ecopark of Borj-cédria. Hammam-Lif. Tunisia.

### 2.2 Determination of total phenolic content

The total polyphenols phenolic compounds were determined according to the method [1]. The results were expressed as gallic acid equivalents (mg GAE/g DR).

### 2.3 Determination of total flavonoid content

The level of flavonoids is determined based on the capacity of formation of a yellow flavonoid-aluminum complex whose maximum absorbance is at 510 nm [2]. The amount of total flavonoid was reported as rutin equivalents (mg RE/g DR).

### 2.4 Condensed tannins contents

The determination of the condensed tannins in the different extracts is carried out according to the method of Broadhurst and Jones [3], modified by Heimler et al. [4]. Condensed tannins were expressed in milligrams of catechin equivalent per gram of extract (mg CE/g DR).

### 2.5 Antioxidant activity

The antioxidant activities were measured by different tests; the scavenging activity on DPPH radical of extracts was estimated as reported by Okawa et al. [5]. The reducing power of the extracts was determined according to the method reported by Choi et al. [6]. The peroxidation of linoleic acid was determined according to the method of Tlili et al. [7].

### 2.6 Antimicrobial activity

The antimicrobial activity of the extracts was determined by the diffusion method in agar medium cited by Oyaizu [8] and Celiktas et al. [9] with a slight modification, this method was employed to determine inhibition diameter of the extract against 6 g negative and gram positive strains.

## Funding sources

This work is part of a Ph.D. thesis at laboratory of Aromatic and Medicinal Plants, Center of Biotechnology at the Ecopark of Borj-cédria. BP-901, 2050 Hammam-Lif. Tunisia.
