# Supplementary material for: Chemical composition, antimicrobial and antioxidant activities data of three plants from Tunisia region: Erodium glaucophyllum, Erodium hirtum and Erodium guttatum
Source: Data Brief. 2018 Jul 10;19:2352–5. doi: 10.1016/j.dib.2018.07.005 (PMC6141786; doi:10.1016/j.dib.2018.07.005)
Supplement: Supplementary file 1 — Supplementary material. [file mmc1.docx]

Subject: Submission of manuscript

Dear Sir,

In this paper we tried to study the phytochemical content (total phenolic compounds, total flavonoids, condensed tannins) and the antioxidant potential and the antimicrobial activities of plant in the Mediterranean genus ***Erodium glaucophyllum, Erodium hirtum* and *Erodium guttatum*** from the Tunisia region.

Best regards,

Gadhoumi Hamza
